# An unexpected tumor-resistant phenotype from floxing PAK1 in a mouse model of colitis associated cancer

**DOI:** 10.1038/s41598-025-12082-8

**Published:** 2025-08-09

**Authors:** Kristine Jimenez, Lambert Lindeck-Pozza, Adrian P. Frick, Maximilian Baumgartner, Felix Haller, Christina Gmainer, Anita Krnjic, Anton Klotz, Manuela Jambrich, Thomas Köcher, Vineeta Khare, Christoph Gasche

**Affiliations:** 1 Division of Gastroenterology and Hepatology, Department of Internal Medicine III, Medical University of Vienna, Waehringer Guertel 18-20, A-1090 Vienna, Austria; 2 CeMM Research Center for Molecular Medicine of the Austrian Academy of Sciences, Vienna, Austria; 3 Vienna Biocenter Core Facilities, Vienna, Austria

**Keywords:** Inflammatory bowel disease, Colitis-associated cancer, Cre-LoxP, AOM/DSS, P-21 activated kinase 1, PAK1, IL10, Colorectal cancer, Inflammatory bowel disease, Colorectal cancer, Cancer genetics

## Abstract

Inflammatory bowel disease (IBD) and colitis-associated cancer are associated with activation of PAK1 (p-21 activated kinase 1). We previously found that total knockout of PAK1 (PAK1KO) reduced tumorigenesis upon AOM/DSS but enhanced tumorigenesis in another model of IBD with total knockout of IL10 (IL10KO). To better understand the specific role of epithelial PAK1, we crossed *Pak1* floxed (PAK1fl) with VillinCre mice for a conditional knockout of PAK1 in intestinal epithelia (PAK1CKO). PAK1fl were included as additional controls. Unexpectedly, inflammation and tumorigenesis were greatly reduced in PAK1fl compared to WT or PAK1KO after AOM/DSS treatment. PAK1CKO had higher tumor incidence and counts compared to PAK1fl, but was still lower in comparison to PAK1KO or WT. When crossed with IL10KO mice, PAK1CKO exacerbated the expected hyperproliferative phenotype, resulting in early mouse morbidity. Despite normal *Pak1* mRNA expression in PAK1fl colonic lysates, PAK1 protein expression on immunohistochemistry was higher that WT. Both PAK1fl and PAK1CKO mice were more resistant to shifts in microbiome, and remained clustered together compared to WT or PAK1KO. Altogether, our results suggest that floxing itself may have altered *Pak1* expression, which conferred protection from AOM/DSS carcinogenesis.

**Supplementary Information:**

The online version contains supplementary material available at 10.1038/s41598-025-12082-8.

## Introduction

Inflammatory bowel disease (IBD) is characterized by chronic intermittent inflammation of the gastrointestinal (GI) tract, which increases susceptibility for neoplasia. In IBD, the complex interplay of genetic susceptibility, environmental stimuli, and host-gut microbiota interactions that maintain intestinal homeostasis is perturbed^1^.

In previous studies, we identified p-21 activated kinase 1 (PAK1) as a common mediator of signaling pathways altered by mesalamine, the mainstay treatment in mild to moderate colitis^2,3^. PAK1 is a member of a family of serine/threonine kinases and effectors of the small Rho-GTPases (Rac1/Cdc42) that participates in diverse cellular processes. In the GI tract, PAK kinases play an important role in disease pathophysiology^4^. PAK1 expression is increased in IBD and colorectal cancer (CRC), as well as several other cancer types. This upregulation promotes cell survival and contributes to oncogenic signaling pathways^5^.

A variety of pre-clinical mouse models have been developed to study IBD and colitis-associated cancer (CAC), inducing colitis by using chemical, genetic, or immune cell-mediated means. Of these, chemical induction using DSS is the most widely used^6^. We have found that complete absence of PAK1 reduced tumor multiplicity without affecting tumor size after azoxymethane (AOM)/DSS treatment. This suggested that PAK1 may have a role in tumor initiation^7^. However, when combined with a model of IBD with total loss of IL10 (IL10KO), this complete absence of PAK1 resulted in an unusual phenotype with hyperproliferation of crypts, and enhanced inflammation and tumorigenesis^8^. These data indicated a fundamental role of PAK1 in intestinal crypt homeostasis and GI pathophysiology. Although we had primarily been utilizing total PAK1 knockout mice, it was clear that to better understand the role of PAK1 in IBD and CRC we would need intestinal epithelial-specific PAK1 deletion.

The Cre-LoxP system is a powerful tool and widely used tool for engineering conditional genetic expression. We crossed *Pak1 flox/flox* (PAK1fl) mice^9^ with *VillinCre* mice^10^ in order to generate a *Pak1* conditional knockout (PAK1CKO) mouse with intestinal epithelial-specific deletion of *Pak1*. Contrary to our expectations, we found that PAK1fl mice did not behave as wild type control mice (WT). Floxing of *Pak1* alone was protective in AOM/DSS, reducing inflammation and tumorigenesis in comparison to total PAK1 knockout mice (PAK1KO) or WT mice.

## Results

### Floxing of Pak1 is protective in mouse models of colitis

**Fig. 1:**
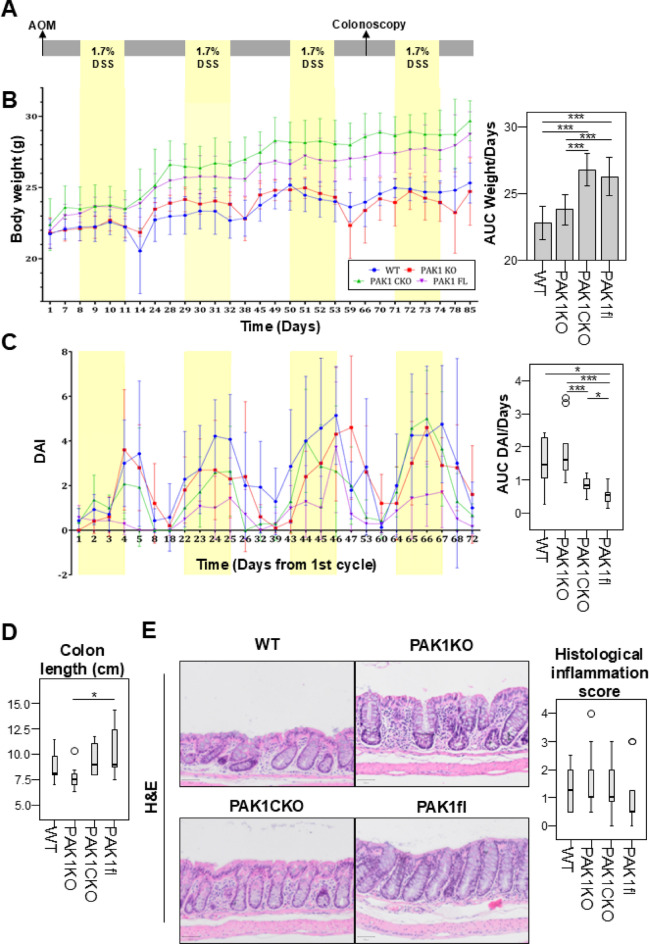
Inflammation is reduced upon floxing of PAK1 A. Treatment Regimen. WT (n=7), PAK1KO (n=9), PAK1CKO (n=11) and PAK1fl (n=11) mice were injected with 10mg/kg BW Azoxymethane and underwent four cycles of 1.7% DSS in the drinking water for four days with 2-week recovery periods. B. Weight curve and calculated area under the curve (AUC) across the experiment divided by the number of days in the experiment. C. Disease activity index (DAI) across the experiment and calculated AUC divided by the number of days in the experiment. D. Native colon lengths at sacrifice. E. Representative histological images of hematoxylin and eosin stains of intestinal epithelia and evaluated histological inflammation score. *p ≤ 0.05, ** ≤ 0.01, *** ≤ 0.001.

In this study, total loss of (PAK1KO) was compared with specific loss of *Pak1* in intestinal epithelium (PAK1CKO) in an AOM/DSS mouse model of CAC (Fig. 1 A). *Pak1* floxed (PAK1fl) as well C57BL/6 (wild type, WT) mice were included as controls.

Contrary to our expectations, clinical markers of inflammation such as weight loss and disease activity index (DAI), which additionally includes stool consistency and rectal bleeding, were significantly worse in WT and PAK1KO animals as compared to PAK1CKO and PAK1fl (Fig. 1B, C). Furthermore, two WT animals were euthanized early due to weight loss. On sacrifice, colon lengths were found shorter in PAK1KO mice in comparison to PAK1fl (Fig. 1D). Histological inflammation did show a trend for PAK1fl to be less inflamed than the other genotypes (Fig. 1E) despite a recovery period between the last DSS cycle and mouse sacrifice.

We also evaluated inflammation in an acute model of colitis, where only one cycle of DSS was administered prior to sacrifice. In this experiment we had not included WT mice, as we were not initially aware that floxed *Pak1* mice were phenotypically different from WT mice. In this short model of colitis, one can see a similar trend where PAK1CKO and PAK1fl had better clinical parameters with less weight loss and bleeding, as compared to PAK1KO (Supplementary Fig. 1 A). This trend was also present on histology, where PAK1fl had the least inflammation and the longest colons (Supplementary Fig. 1B-D).

Our group previously discovered that crossing PAK1KO with *IL10* knockout (IL10KO) mice, a mouse model that develops spontaneous colitis, resulted in a hyperproliferative phenotype with increased inflammation and tumorigenesis^8^. We intended to cross PAK1CKO into IL10KO (IL10CKO, Supplementary Fig. 3 A), however could not establish a stable breeding lineage. IL10CKO mice would become ill at 3–4 months, in comparison to PAK1KO crossed with IL10KO (IL10DKO) mice which could be healthy up to 10 months. We examined a few IL10CKO mice and found that, as early as 11 weeks, mice colons were already hyperproliferative, with drastically thickened epithelia and resultant shortened colons in comparison to IL10DKO (Supplementary Fig. 2A-D). Unfortunately, we did not have IL10KO x PAKfl mice for comparison. At 18 weeks, some of the sampled IL10DKO mice would also develop this hyperproliferative phenotype. Hyperproliferation was however absent in IL10KO, PAK1KO, PAK1CKO, and PAK1fl mice (Supplementary Fig. 2D). Thus, while mice with floxed *Pak1* were protected from AOM/DSS, this was not the case in an IL10KO model of IBD.

### Pak1 expression is increased in floxed mice

**Fig. 2:**
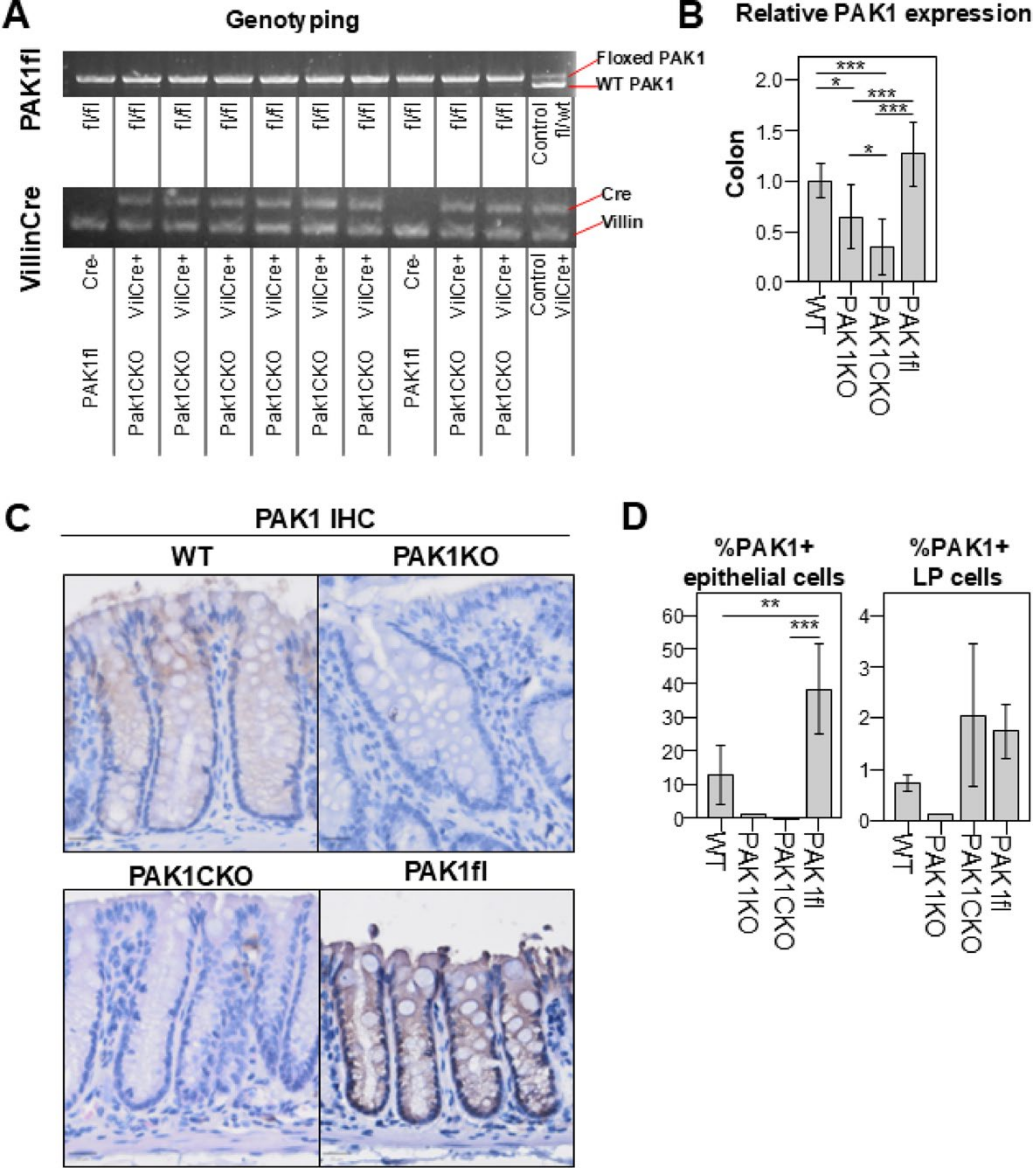
PAK1 protein expression is increased in floxed mice A. Cropped representative PCR blots from tail genotyping for floxed PAK1 and VillinCre, edited to increase contrast (Original images in Supplementary Figure 3B) B. Relative expression of PAK1 in whole colon lysates from untreated animals WT (n=3), PAK1CKO (n=3), PAK1fl (n=3). Normalized to Actin. C. Representative images of PAK1 staining in colons from AOM/DSS-treated mice D. Mean percentage of PAK1 positive cells in epithelial cells and lamina propria (LP) WT (n=4), PAK1CKO (n=5), PAK1fl (n=4). PAK1KO is only included on the graph as a reference and is not included in statistics ** ≤ 0.01, *** ≤ 0.001.

Due to the unexpected phenotype of PAK1fl mice, we examined colonic PAK1 expression. Prior the experiments, we selected mice based upon their genotype using standard PCR. Re-genotyping from tail tissue post-mortem was as expected: PAK1fl mice were flox/flox for *Pak1* and lacking *Cre*, while PAK1CKO were both flox/flox and *Cre*-positive (Fig. 2 A, Supplementary Fig. 3B). PAK1KO mice likewise possessed no WT allele and both copies of *Pak1* were knocked out (Supplementary Fig. 3 C). *Pak1* expression was evaluated using quantitative real-time PCR (qPCR) on whole colon isolates and whole small intestinal lysates and as expected was found to be reduced in both PAK1KO and PAK1CKO in comparison to WT (Fig. 2B, Supplementary Fig. 3D), but was similar between PAK1fl and WT. Upon immunohistochemical staining, however, we were surprised to find that PAK1 expression was actually increased in PAK1fl mice (Fig. 2 C). We used HALO v3.4 to quantify PAK1 expression in colonic crypts and lamina propria. As expected, there was no PAK1 expression in PAK1KO or PAK1CKO epithelia. However, PAK1 expression in epithelial cells was higher in PAK1fl as compared to WT (Fig. 2D). In the lamina propria (LP), PAK1 expression was absent in PAK1KO but seemed to be higher in PAK1fl mice as compared to WT. This suggests that the increased PAK1 expression is mediated at the protein level.

### Floxing of Pak1 protects from AOM/DSS tumorigenesis

**Fig. 3:**
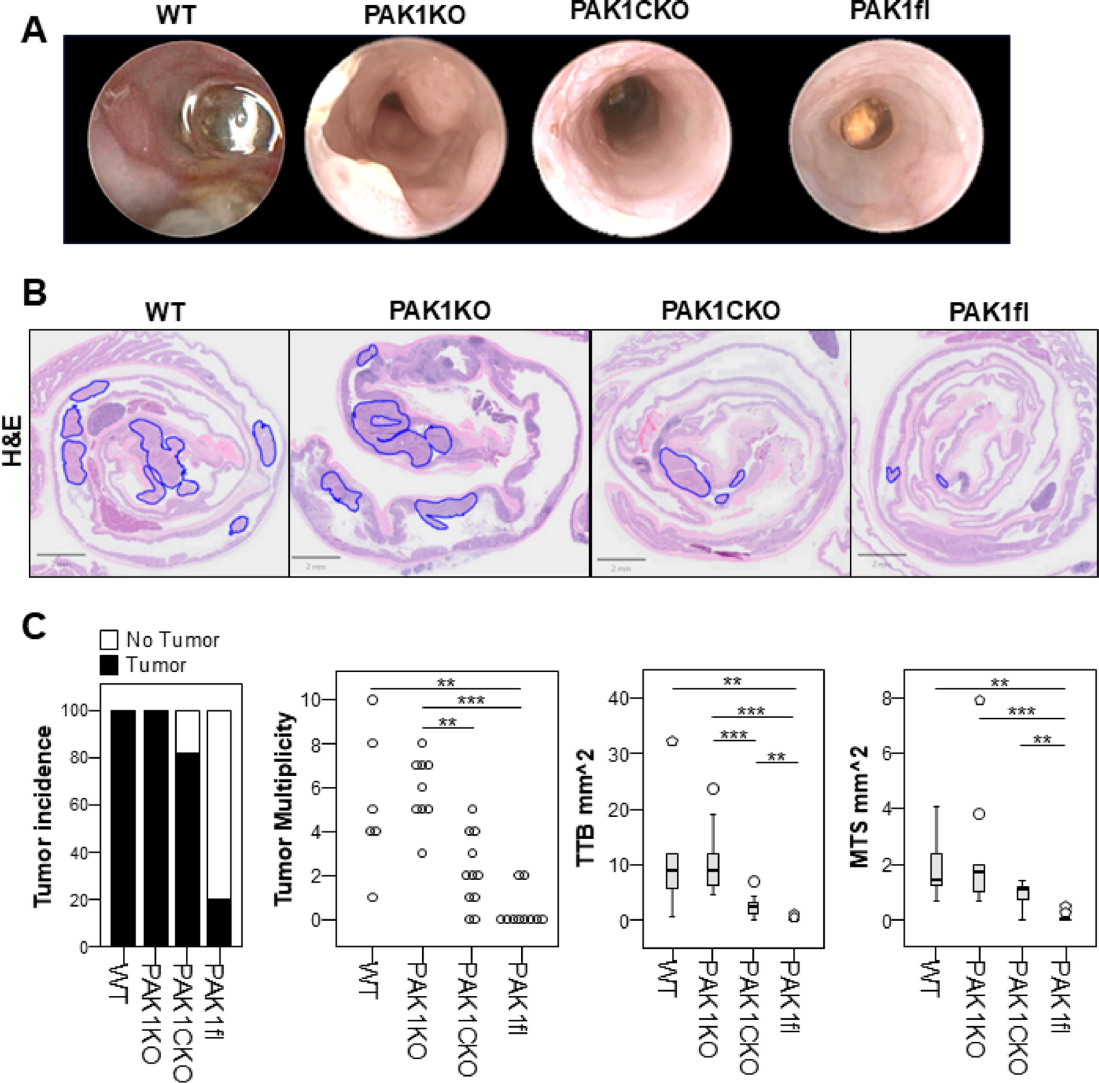
Floxing of PAK1 protects against AOM/DSS tumorigenesis A. Representative colonoscopy images from colonoscopy performed after the 3rd cycle of DSS. B. Representative histological images of swiss-rolled colons, with tumors outlined in blue C. Tumor incidence, multiplicity, total tumor burden, and mean tumor size. ** p ≤ 0.01, *** ≤ 0.001 WT (n=6), PAK1KO (n=9), PAK1flVilcre (n=11), PAK1fl (n=10).

After three cycles of DSS in the AOM/DSS experiment, animals underwent colonoscopy to assess tumor development. At this timepoint, there were clearly more visible tumors in WT and PAK1KO mice as compared to PAK1CKO and PAK1fl mice (Fig. 3A). This was corroborated by histology (Fig. 3B). Tumor incidence was reduced by 80% in PAK1fl and 20% in PAK1CKO as compared to WT or PAK1KO (Fig. 3C). Tumor multiplicity, total tumor burden, and the mean tumor size were also reduced in PAK1fl and PAK1CKO as compared to WT or PAK1KO, likewise with greater reductions in PAK1fl as compared to PAK1CKO. We also examined PAK1 protein expression in tumor tissue and found that staining intensity was higher in both WT and PAK1fl tumors as compared to normal tissue (Supplementary Fig. 4). PAK1KO and PAK1CKO tumor tissue did not have any appreciable differences in staining to their non-tumor counterparts. The very low number of tumors in PAK1fl precluded any meaningful statistical analysis.

In summary, despite the inclusion of PAK1fl as a control and the expectations of a similar phenotype as WT mice, this genotype was more resistant to AOM/DSS-induced colitis and tumorigenesis. Loss of epithelial PAK1 in the PAK1CKO mice diminished this protective effect, but did not reach the level of WT or PAK1KO.

### Floxing of PAK1 confers a microbial profile that is more resistant to shifts upon AOM/DSS

**Fig. 4:**
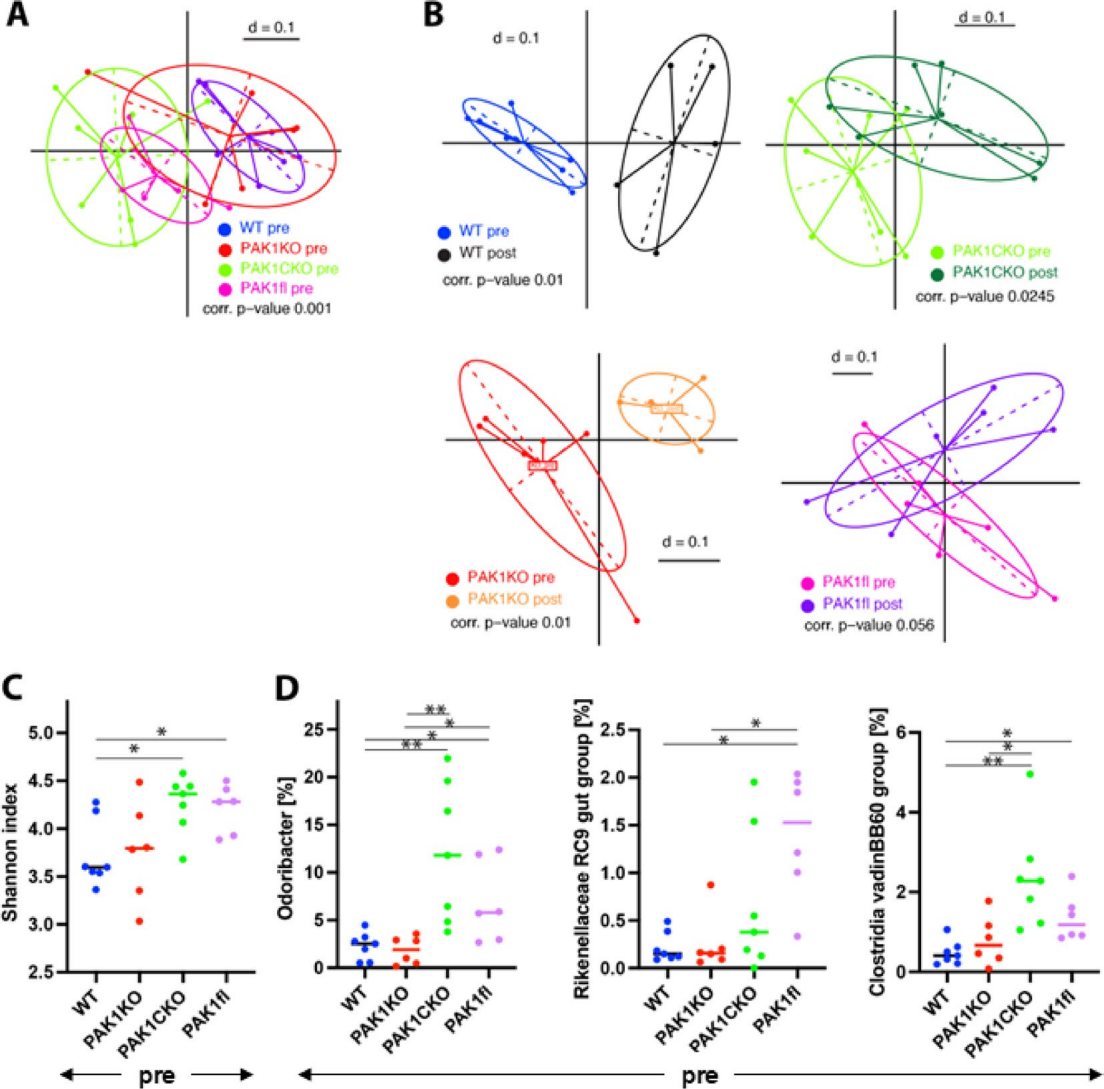
Genotype co-housing and AOM/DSS treatment do not shift fecal microbial profiles in PAK1fl mice A. NMDS plot of fecal microbial profiles after co-housing genotypes, but prior to DSS treatment (pre-) B. NMDS plots comparing fecal microbial profiles in pre- versus post-AOM/DSS treatment. C. Shannon index of pre- samples. D. Differentially expressed genera in pre- samples of floxed PAK1 versus WT or PAK1KO mice. WT (n=7), PAK1KO (n=6), PAK1CKO (n=7) and PAK1fl (n=6) * ≤ 0.05, ** ≤ 0.01.

Genotypes were mixed for 2 weeks prior to experiments to homogenize microbiome, and were then divided according to genotype for the experiment proper. Fecal samples were collected after the mixing but prior to experimentation (hereafter referred to as pre) as well as after AOM/DSS treatment (post). On fecal microbiome analysis, WT and PAK1KO clustered together in NMDS plots, while PAK1CKO and PAK1fl formed their own separate cluster (Fig. 4A). This pattern was maintained in fecal samples collected after AOM/DSS treatment (Supplementary Fig. 5 A).

NMDS plots comparing pre- and post-AOM/DSS samples within genotypes revealed that AOM/DSS treatment shifted microbial profiles in WT, PAK1KO and to a lesser extent in PAK1CKO, but not in PAK1fl (Fig. 4B). Shannon diversity was higher in PAK1CKO and PAK1fl pre- samples (Fig. 4C). Several bacterial genera were found to have a higher relative abundance in pre-PAK1CKO and/or PAK1fl (Fig. 4D) but were different from those found to be differentially abundant in post-AOM/DSS samples (Supplementary Fig. 5B). Bile acid analysis was also performed on these fecal samples but no differences were found across genotypes in pre- and post-AOM/DSS samples (Supplementary Fig. 5 C). Supplementary Table 3 lists the bile acids analyzed and the corresponding measurements before and after DSS treatment.

## Discussion

PAK1 is overexpressed in human IBD and CRC^5,11,12^and we have previously shown that total *Pak1* deletion in a mouse model of IBD reduces tumor multiplicity upon AOM/DSS treatment^7^. However, in IL10KO mice, another mouse model of IBD, *Pak1* deletion exacerbates colitis and tumorigenesis^8^. In this study we crossed *Pak1* floxed mice^9^ with *VillinCre* mice^10^ for an intestinal epithelial-specific deletion of *Pak1* as we had previously been using total knockouts. We were surprised to find that floxing of *Pak1* alone reduced inflammation in both chronic and acute models of DSS colitis. Tumorigenesis was also greatly reduced in PAK1fl mice, which was mildly reversed in PAK1CKO, but tumor counts and size were still greatly reduced in comparison to WT or PAK1KO. In IL10KO mice, however, we found that PAK1CKO exacerbated the expected phenotype, resulting in early mouse morbidity. Examination of PAK1 expression revealed that despite having the expected genotype and *Pak1* expression on qPCR, PAK1 expression on immunohistochemistry was higher in *Pak1* floxed tissues. This suggests that floxing itself may have altered *Pak1*, which conferred protection from colitis and tumorigenesis upon AOM/DSS, but not from colitis upon total loss of IL10. Furthermore, microbiome analysis also revealed that both PAK1fl and PAK1CKO mice were more resistant to shifts in microbiome, and remained clustered together despite mixing with other genotypes and despite treatment with AOM/DSS.

Previously, our group found that loss of PAK1 impaired intestinal tumorigenesis based on results from mice with a mutated APC allele, as well as an AOM/DSS model. In particular, AOM/DSS treatment in PAK1KO was found to reduce tumor number but not tumor size in in comparison to WT mice^7^.In this study both tumor number and size were similar between WT and PAK1KO, albeit tumor size being measured by colonoscopy in the previous study and by histology in the current study. This discrepancy could be due to differences in sample size, which is smaller in the current study, but also due to differences in tumor analysis. The difference in tumor number in the previous study was primarily due to reduction in dysplastic lesions, which were not separately analyzed in this study. When comparing carcinoma counts, results were similar in PAK1KO and WT which is comparable to what was seen in this study.

Despite expectations that PAK1fl would serve as a control like WT, there was a drastic reduction in weight loss and disease activity upon AOM/DSS treatment in comparison to WT mice. Furthermore, tumor incidence, multiplicity, and size were also greatly reduced in Pak1fl. While there are studies that show off-target effects when using Cre-recombinase, in our mice the changes were observed in Pak1fl mice that did not have Cre-recombinase. We theorized that PAK1 expression may have been altered by floxing, and indeed found a higher expression of PAK1 in PAK1fl colons in comparison to WT in both epithelial and LP regions by IHC. Epithelial PAK1 was absent in PAK1CKO, but PAK1 expression in LP was still higher than in WT. Given that mRNA expression was not higher in PAK1fl, we theorize that these changes occur at the protein level. However, it must be noted that qPCR was performed on whole intestinal lysates from untreated animals, and changes in PAK1 may be primarily upon treatment with AOM/DSS. It is also not clear why PAK1 expression in PAK1CKO is reduced in comparison to PAK1KO. A potential cause would be if the mRNA expression of actin, the endogenous control, was modulated in PAK1fl mice. While PAK1 does not directly regulate Actin transcription, it is known that PAK1 modulates cytoskeletal remodeling, facilitating cell migration and invasion when overexpressed in cancer^4,13,14^. PAK1 inducing actin polymerization could indirectly promote Actin transcription by reducing the pool of actin that sequesters MAL, allowing it to interact with the transcription factor SRF^15,16^. If this is the case, then increased actin transcription could mask changes on qPCR in the PAK1fl background.

Floxing alone can result in unintended effects; one study found that otherwise healthy Pax6 floxed mice had an abnormal eye phenotype that was significantly different from wild type Pax6 mice^17^. The PAK1fl mice used in our study were originally generated for cardiomyocyte-specific deletion of PAK1. However, Liu, et al. did not use wild type mice for comparison and no aberrant PAK1 expression was reported^9^. To our knowledge, other studies using this PAK1fl mice either did not use a wild type control, or did not look into PAK1 expression so aberrant expression could have been overlooked^18–20^. It is also possible that the alterations in PAK1 are only relevant in this particular organ system or disease context.

It is not clear how floxing has altered PAK1 expression. PAK1 regulation is mediated through phosphorylation, which depending on the phosphorylation site influences protein stability, substrate interaction, as well as degradation^21^. Thus, alteration at of these sites by floxing could influence PAK1 stability or degradation, allowing cellular accumulation. Furthermore, it is also possible that the alteration is not only quantitative, but also qualitative, where PAK1 function is amplified. Further studies are needed to determine how floxing has altered PAK1, in structure or in function.

Apart from the previous study where PAK1 loss impaired tumor initiation in AOM/DSS models^7^our group also had a study where we crossed PAK1KO with IL10KO, a mouse model of IBD which develops spontaneous colitis. There, loss of PAK1 exacerbated colitis and tumorigenesis, displaying a distinct intestinal epithelial hyperproliferative phenotype with elongated crypts and loss of goblet cells^8,22^. We attempted to cross PAK1CKO into IL10KO mice, but were ultimately unable to produce a stable breeding line as mice would become ill very early, in comparison to IL10DKO mice which would be stable well into late adulthood. We collected tissue from a few animals with the correct IL10CKO genotype, and found earlier development of hyperproliferation with loss of goblet cells, which could explain their poor survival. IL10DKO mice had reduced survival in comparison to IL10KO previously^8^.

PAK1 is involved in a variety of pathways including cytoskeletal remodeling, response to oxidative stress, cell proliferation and survival. It is overexpressed in variety of cancer types, and can promote carcinogenesis from initiation to metastasis^13,21^. In our study, the inadvertent increased expression of PAK1 as a result of floxing conferred protection from AOM/DSS itself. When *Pak1* is removed from intestinal epithelial cells in PAK1fl mice (PAK1CKO), the protective effect is reduced, albeit not completely abrogated as multiplicity and size were still lower in comparison to PAK1KO or WT. Thus, both epithelial and non-epithelial floxed PAK1 may have protective functions in the AOM/DSS model. Conversely, when PAK1CKO is crossed into the IL10KO background, it exacerbates the hyperproliferation and tumorigenesis that is seen in IL10KO crossed with PAK1KO mice. This suggests that it is the loss of PAK1 in epithelial cells that is responsible for the phenotype, and that non-epithelial PAK1 may further contribute to the phenotype. Unfortunately, we did not have PAK1fl x IL10KO mice for comparison.

One potential protective role of PAK1 could be promoting the response to oxidative stress, countering DNA damage caused by AOM/DSS. PAK1 could also promote cell proliferation and cell survival, allowing faster epithelial regeneration after DSS. PAK1 promoting cell motility could also contribute to repair of denuded areas^16^. Our group has previously shown that overexpression of PAK1 in the normal colonic epithelial cell line HCEC-1CT promoted cell proliferation and reduced apoptosis^5^. However, PAK1 is overexpressed in epithelia during DSS colitis, and PAK1 is overexpressed in IBD and CAC, and is not protective in this context^23^. Perhaps it is a matter of timing, and PAK1 overexpression is protective prior to the damaging event, or just in this specific model of colitis.

Considering the potential non-epithelial components, of interest would be PAK1 overexpression in immune cells. In cancer, PAK1 is involved in facilitating immune evasion^13^and does so by modulating immune surveillance. For example, in pancreatic ductal adenocarcinoma, inhibition of PAK1 increased intratumoral cytotoxic lymphocytes, augmenting cancer cell death^24^. In that study, inhibition of PAK1 downregulated PD-L1, an immune checkpoint ligand which suppresses anti-tumor immunity. PAK1’s role in cytoskeletal dynamics could also affect immune cell migration and function. For example, PAK1 influences the directional migration of neutrophils^25^vital for innate immunity and characteristically increased in active IBD. Modulation of the immune response upon AOM/DSS colitis may be a further mechanism that PAK1 overexpression could be protective in our study. In IL10KO, PAK1 overexpression in non-epithelial cells combined with the loss of PAK1 in epithelial cells led to a more severe phenotype. It could be that the much higher levels and continuous oxidative stress in this model and the lack of anti-inflammatory IL10 outweighed any potential protective effects.

Apart from altered PAK1 expression, another potential factor could be differences in microbiome. Previous studies have shown that gut microbiota can influence outcomes in AOM/DSS, and that more diverse microbiomes are protective^26,27^. Co-housing has been shown to be a viable means to homogenize microbiome^28^. However, we found that despite genotype mixing, PAK1KO and WT mice clustered together while PAK1CKO and PAK1fl formed a separate cluster. Shannon diversity was also higher in PAK1fl and PAK1CKO mice as compared to PAK1KO and WT at baseline. AOM/DSS treatment altered microbial profiles in WT, PAK1KO, and to a lesser extent PAK1CKO, but not in PAK1fl. This mirrors the pattern seen in tumor parameters; however, it is difficult to say if the alterations in microbial profile is a contributor or a response to tumorigenesis. We hypothesize that floxing of PAK1 may be enforcing a particular microbial profile, however it may be that a longer co-housing period is necessary to homogenize microbiota. This was not possible in our experiment as regrouping by genotype would not work in older, more aggressive male mice. Another study recommends strain intercrossing to fully homogenize microbiome across the gastrointestinal tract, but this would be logistically complex and likely not feasible for four genotypes^29^.

Our initial aim was to generate a mouse model with a specific loss of epithelial PAK1, as PAK1 has multiple functions and is expressed in a variety of cell types. We intended to determine if the observations that we had in our previous studies were due to intestinal epithelial PAK1 and chose a PAK1fl mouse that had been used in previous publications. We found that PAK1fl mice did not, as one would expect, behave like WT mice upon AOM/DSS treatment. Had we omitted either WT or PAK1fl mice under the assumption they were similar, we would have had vastly different conclusions. While these differences may be model or organ-system dependent, future studies using this model must take into consideration that there may be issues with PAK1 expression or function and ignoring such issues would lead to incorrect interpretation of data. While it is interesting that these issues have such a strong effect upon tumorigenesis, this model cannot serve the purpose for which we intended, and should not be run without including further appropriate controls like a wild type group. Even then, it would be complex to dissect if the effects are due to increased PAK1 expression in PAK1fl-non-deleted cells or actually due to specific PAK1-deletion. In summary, we found that floxing of PAK1 unexpectedly increased PAK1 expression, which conferred protection against inflammation and tumorigenesis in an AOM/DSS model.

## Methods

### Ethics statement

Ethical approval for all animal experiments was obtained from the Austrian Federal Ministry of Education, Science and Research (GZ: BMWFW-66.009/0324-WF/V/3b/2016). All experiments were conducted in compliance with Austrian and European law and Good Scientific Practice guidelines of the Medical University Vienna.

This study is reported in accordance with the ARRIVE guidelines.

### Animal studies

*Pak1* floxed (PAK1fl) mice^9^ were crossed with *VillinCre* mice^10^ for conditional loss of PAK1 in intestinal epithelium (PAK1CKO, *n* = 11). These mice were compared with PAK1 knockout (PAK1KO, *n* = 9), and C57BL/6 wild type (WT, *n* = 7) and PAK1fl (*n* = 11) mice as controls. For list of mouse strains used see Supplementary Table 2.

After an initial adaptation phase of 2 weeks, during which genotypes were mixed to homogenize microbiome, 6 to 8-week-old male mice were separated by genotype into cages and then injected with 10 mg/kg BW Azoxymethane i.p. (AOM, Sigma) to induce DNA damage (Fig. 1 A). Animals were then subjected to 4 cycles of 1.7% Dextran sodium sulphate (DSS) in their drinking water for four days followed by 2 weeks of recovery with plain water *ad libitum*. All animals were housed at the Center for Biomedical Research and Translational Surgery, Medical University of Vienna and kept under 12-hour light/dark cycles. Standard chow was available *ad libitum*.

Clinical signs of inflammation such as percentage weight loss, stool consistency, and presence of blood in stool were scored and summarized in a disease activity index (DAI) as previously described^8^. The presence of blood in stool was evaluated using a Guajak-based test (HemoCARE). DAI was measured daily during DSS cycles, and weekly otherwise.

At the end of the experiment, animals were euthanized by initial i.p. injection with an overdose of Ketamine and Xylazine followed by cervical dislocation. Intestines were collected, stool collected from colons and snap-frozen. Intestines were flushed with PBS; lengths were measured and a small section of colon was put into RNAlater (Sigma) and snap-frozen for later processing. Remaining material was swiss-rolled, fixed in 10% formalin and processed into paraffin blocks. Histological inflammation was scored by two independent examiners, Dr. Adrian Frick and Lambert Lindeck-Pozza, on H&E-stained sections as described previously^30^. Whole slides were scanned (Olympus SLIDEVIEW V200) and tumor parameters analyzed using QuPath^31^. Tumor incidence is the percentage of mice of each genotype that developed tumors, multiplicity is the count of tumors per mouse, total tumor burden (TTB) is the summative areas of all tumors per mouse, and mean tumor size (MTS) is the TTB divided by the tumor count.

For the acute DSS model, eight-week-old PAK1KO (*n* = 6), PAK1CKO (*n* = 8), and PAK1fl (*n* = 5) mice were given 1.7% DSS in drinking water for 6 days, followed by 2 days of plain water and subsequent termination. Weight loss and bleeding were monitored daily. Intestinal tissue was swiss-rolled and processed into paraffin blocks.

IL10KO mice were graciously provided by Dr. Terrence Barrett (University of Kentucky, Lexington, KY) and Jeffrey B. Brown (Northwestern University, Feinberg School of Medicine, Chicago, IL). We had previously crossed PAK1KO into IL10KO (IL10DKO)^8^and intended to create PAK1CKO x IL10KO (IL10CKO) but could not form a stably breeding lineage. Mice used in this study were those of the correct genotype arising sporadically. For a list of the mice and their backgrounds used in this study see: Supplementary Table 2.

### Genotyping

Genotyping was performed on toe clippings, ear punch material, or post-mortem tail clippings. For primer list see Supplementary Table 1.

### Colonoscopy

At 8 weeks, after the third DSS cycle, colonoscopy was carried out in small batch of animals but was discontinued due to animal deterioration. Animals were fasted and given preparatory solution 24 h prior to colonoscopy (20 g glucose, 2.9 g trisodium citrate dihydrate, 2.6 g NaCl, 1.5 g KCl in one liter water) supplied with Polyethyleneglycol (34.5 g/L PEG 4000). Animals were anesthetized (180 mg Ketamine/g BW, 3.6 mg Xylazine/g BW in 0.9%NaCl), and colonoscopy performed using an endoscope (Karl Storz Xenon Nova 175) connected to a camera system (Karl Storz Telecam SL II 202121).

### Immunohistochemistry

Sections were deparaffinized and rehydrated, and endogenous peroxidase was blocked (BLOXALL, Vector Laboratories). Antigen retrieval was performed in heated citrate buffer (Dako), and slides were incubated with 1:100 PAK1 primary antibody (Cell Signaling Technology) overnight at 4 °C. Staining proceeded with application of biotinylated secondary antibody, avidin-biotin-HRP complex (PK-4000, Vectastain), and the DAB chromogen system (Dako) was applied. Slides were counterstained with Hematoxylin.

Whole slides were scanned at 40X (Olympus Slideview VS200) and analyzed with HALO v3.4 using a tissue classifier and the area quantification FL (v2.1.10) or cytonuclear IHC algorithms. The percentage of PAK1-positive nuclei was quantified in at least 50 colonic crypts.

### Quantitative real-time polymerase chain reaction

Whole intestinal tissue was homogenized by bead beating in TRIzol (ThermoFisher Scientific) and isolated according to manufacturer. Complementary DNA was synthesized with the High-Capacity cDNA Reverse Transcription Kit (ThermoFisher Scientific). Quantitative Real-Time PCR (qPCR) was performed using Fast SYBR Green Master Mix (Applied Biosystems) on an ABI Prism 7500 fast (Applied Biosystems) according to manufacturer. Target *Pak1* (QT00152887, Qiagen) was normalized to endogenous *Actin*. Amplification data was processed using LinRegPCR^32^ to calculate starting concentrations.

### Microbiota analysis

Fecal DNA was isolated after an initial bead-beating step in Lysing Matrix E tubes (MP Biomedicals) with a Precellys 24 homogenizer (Bertin instruments) at 5500 rpm 1 × 30 s and then using the QIAamp DNA stool mini kit protocol (Qiagen).

16 S rRNA gene sequencing and raw data processing was performed at the Joint Microbiome Facility of the Medical University of Vienna and the University of Vienna (project IDs JMF-2208-03) as described previously^33^.

​​For the analysis of sample similarity Rhea scripts were used^34^.Briefly, generalized UniFrac distances were visualized using non-metric Multi-Dimensional Scaling (NMDS). Cluster significance was assessed using permutational multivariate analysis of variance. Testing for significant differences in diversity and bacterial abundances was performed using Kruskal-Wallis Rank Sum Test.

### Bile acid analysis

Bile acid analysis was carried out as previously described^35^ Briefly, stool samples were dried in a vacuum centrifuge and ten milligrams of dried stool was weighted into a Lysing Matrix E tube and subjected to bead-beating at 6000 rpm for 3 × 30 s. After homogenization, BAs and their conjugates were quantified using liquid chromatography–tandem mass spectrometry, employing selected reaction monitoring. A table of raw bile acid data has been attached as supplementary data.

### Statistical analysis

Statistical testing was performed using IBM SPSS 23. Shapiro-Wilk was used to test normality. Normally distributed data was tested using One-way ANOVA with Tukey´s post-hoc test. Non-parametric data was tested using Kruskal-Wallis, and Man-Whitney U-tests with Bonferroni correction for between group comparisons.

## Electronic supplementary material

Below is the link to the electronic supplementary material.

Supplementary Material 1

Supplementary Material 2

Supplementary Material 3

## Data Availability

The datasets generated and/or analysed during the current study have been uploaded to the BioProject Database under the Bioproject ID: PRJNA1196364.And when made public will be available at https://www.ncbi.nlm.nih.gov/bioproject/1196364).For the purposes of technical checks we have added a preliminary access link: https://dataview.ncbi.nlm.nih.gov/object/PRJNA1196364?reviewer=68v1mnjpnpgs5ohi62i5tifbigAll authors had access to the study data and had reviewed and approved the final manuscript.
