# Supplementary material for: An unexpected tumor-resistant phenotype from floxing PAK1 in a mouse model of colitis associated cancer
Source: Sci Rep. 2025 Aug 9;15:29174. doi: 10.1038/s41598-025-12082-8 (PMC12335604; doi:10.1038/s41598-025-12082-8)
Supplement: Supplementary file 2 — Supplementary Material 2 [file 41598_2025_12082_MOESM2_ESM.pdf]

Supplementary Tables

Supplementary Table 1. Genotyping primers

Target /Use	Name	Sequence/Source
PAK1fl Genotyping	PAK1fl fw	5‘-GACCTGGGTTAAATCAC-3‘
	PAK1fl rv	5‘-GGTCTGCCCTTTACCAGG-3‘
VillinCre Genotyping (PCR)	Villin1 fw (2kbseqS)	5‘-CAAGCCTGGCTCGACGGCC-3‘
	Villin1 WT rv (16776)	5‘-TATAGGGCAGAGCTGGAGGA-3‘
	Cre rv (198)	5‘-CGCGAACATCTTCAGGTTCT-3‘
PAK1KO Genotyping	PAK1 fw	5'-GCCCTTCACAGGAGCTTAATGA-3'
	PAK1 WT rv	5'-GAAAGGACTGAATCTAATAGCA-3'
	PAK1KO rv	5'-CATTTGTCACGTCCTGCACGA-3'
IL10KO Genotyping	IL10KO rv (oIMR7376)	5'-CCACACGCGTCACCTTAATA-3'
	IL10 fw (oIMR9573)	5'-CTTGCACTACCAAAGCCACA-3'
	IL10 WT rv (oIMR9574)	5'-GTTATTGTCTTCCCGGCTGT-3'

Supplementary Table 2. Mouse Lineages

Lineage	Background	Mutations	Source	Reference
PAK1KO	C57BL/6J	*Pak1* -/-	Mutant Mouse Regional Resource Center, University of North Carolina (031838-UNC)	**McDaniel, et al.** [1]
PAK1fl	C57BL/6J	*Pak1* fl/fl	Dr. Xin Wang (University of Manchester, Manchester, UK) and Dr. Ming Lei (University of Oxford, Mansfield Road, Oxford, UK).	**Liu, et al.** [2]
VillinCre	C57BL/6J	*VillinCre* (tg/wt)	Jackson Laboratories (#021504)	**Madison, et al.** [3]
IL10KO	C57BL/6J	*IL10* -/-	Dr Terrence A. Barrett (University of Kentucky, Lexington, KY) and Jeffrey B. Brown (Northwestern University, Feinberg School of Medicine, Chicago, IL).	**Kuhn, et al.** [4]
WT	C57BL/6J		Laboratory animal science and genetics, Center for Biomedical Research, Medical University of Vienna	

References

1 McDaniel, A. S. *et al.* Pak1 regulates multiple c-Kit mediated Ras-MAPK gain-in-function phenotypes in Nf1+/- mast cells. *Blood* **112**, 4646-4654, doi:10.1182/blood-2008-04-155085 (2008).

2 Liu, W. *et al.* Pak1 as a novel therapeutic target for antihypertrophic treatment in the heart. *Circulation* **124**, 2702-2715, doi:10.1161/CIRCULATIONAHA.111.048785 (2011).

3 Madison, B. B. *et al.* Cis elements of the villin gene control expression in restricted domains of the vertical (crypt) and horizontal (duodenum, cecum) axes of the intestine. *J Biol Chem* **277**, 33275-33283, doi:10.1074/jbc.M204935200 (2002).

4 Kuhn, R., Lohler, J., Rennick, D., Rajewsky, K. & Muller, W. Interleukin-10-deficient mice develop chronic enterocolitis. *Cell* **75**, 263-274, doi:10.1016/0092-8674(93)80068-p (1993).
