# Supplementary material for: An unexpected tumor-resistant phenotype from floxing PAK1 in a mouse model of colitis associated cancer
Source: Sci Rep. 2025 Aug 9;15:29174. doi: 10.1038/s41598-025-12082-8 (PMC12335604; doi:10.1038/s41598-025-12082-8)
Supplement: Supplementary file 3 — Supplementary Material 3 [file 41598_2025_12082_MOESM3_ESM.pdf]

Supplementary Figures

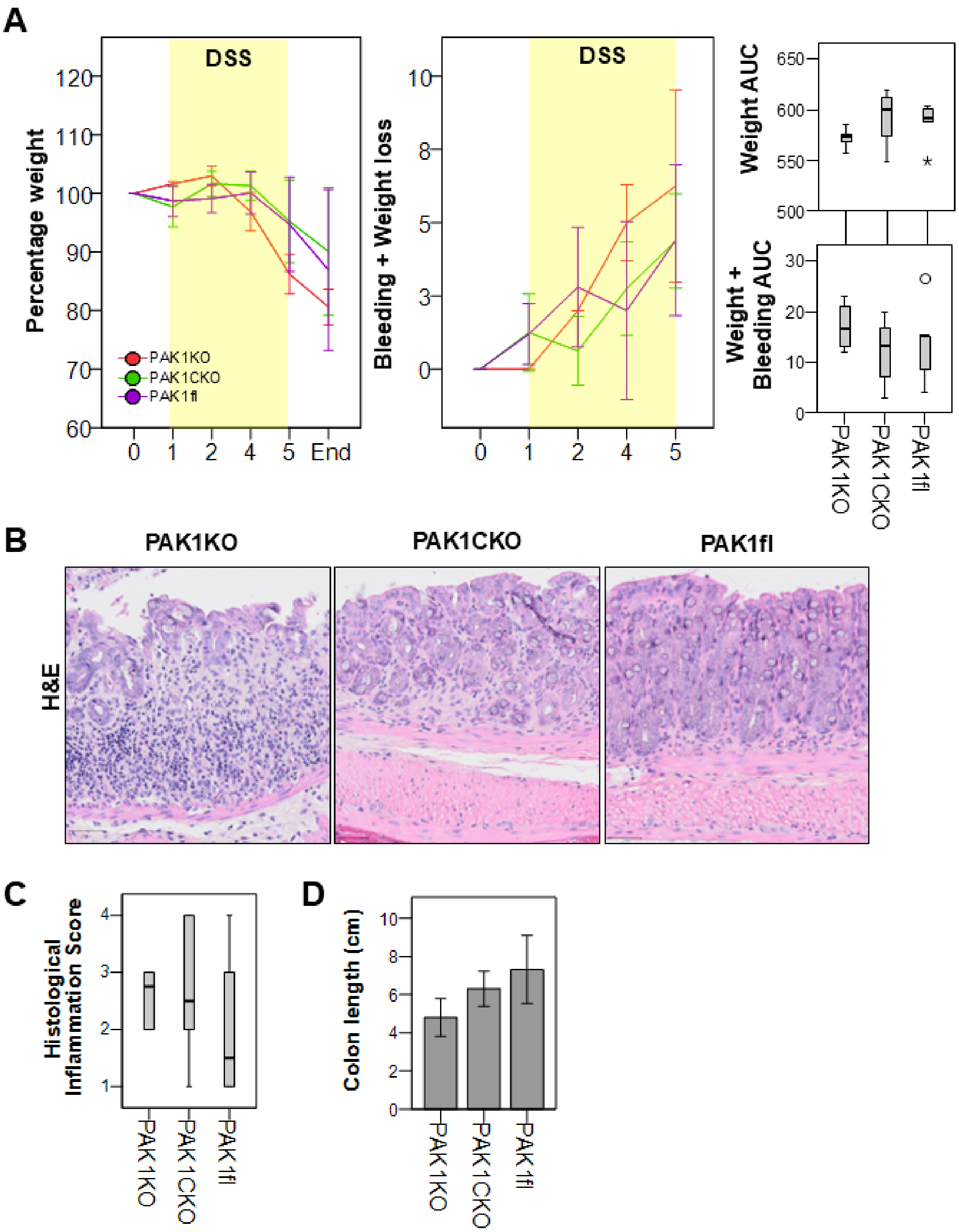

Supplementary Figure 1. Inflammation is reduced in PAK1fl mice in an acute DSS model of colitis. PAK1KO (n=6), PAK1CKO (n=8), and PAK1fl (n=5) treated with 1.7% DSS in drinking water for 5 days, then sacrificed after 2 days. A. Weight as a percentage of the starting weight, Bleeding and weight loss scores as a representative of disease activity, and AUCs of the respective data. B. Representative H&E images of colon C. Histological inflammation score. D. Length of native colon after sacrifice.

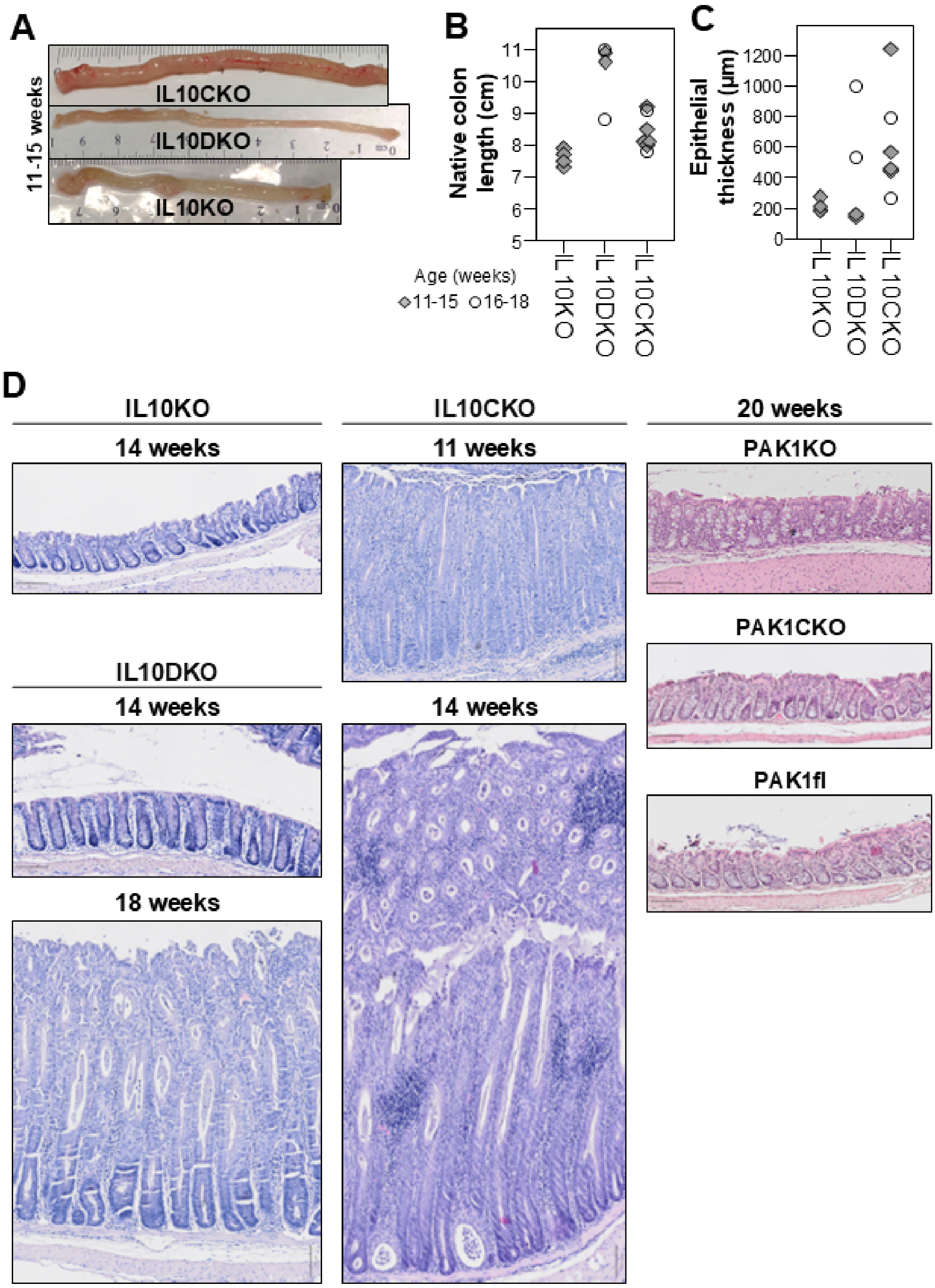

Supplementary Figure 2. Earlier onset of hyperproliferation in PAK1CKO mice Earlier onset of hyperproliferation and spontaneous inflammation on loss of epithelial vs total loss of PAK1 in IL10KO mice. A. Native colons and colon lengths of IL10KO (n=4), DKO (n=4), and IL10CKO (n=6) mice at 11-15 weeks or 16-18 weeks of age. B. Epithelial thickness (μm) measured from histology D. Representative images of H&E-stained colons from IL10KO, IL10DKO, IL10CKO, PAK1CKO, and PAK1fl at varying mouse ages

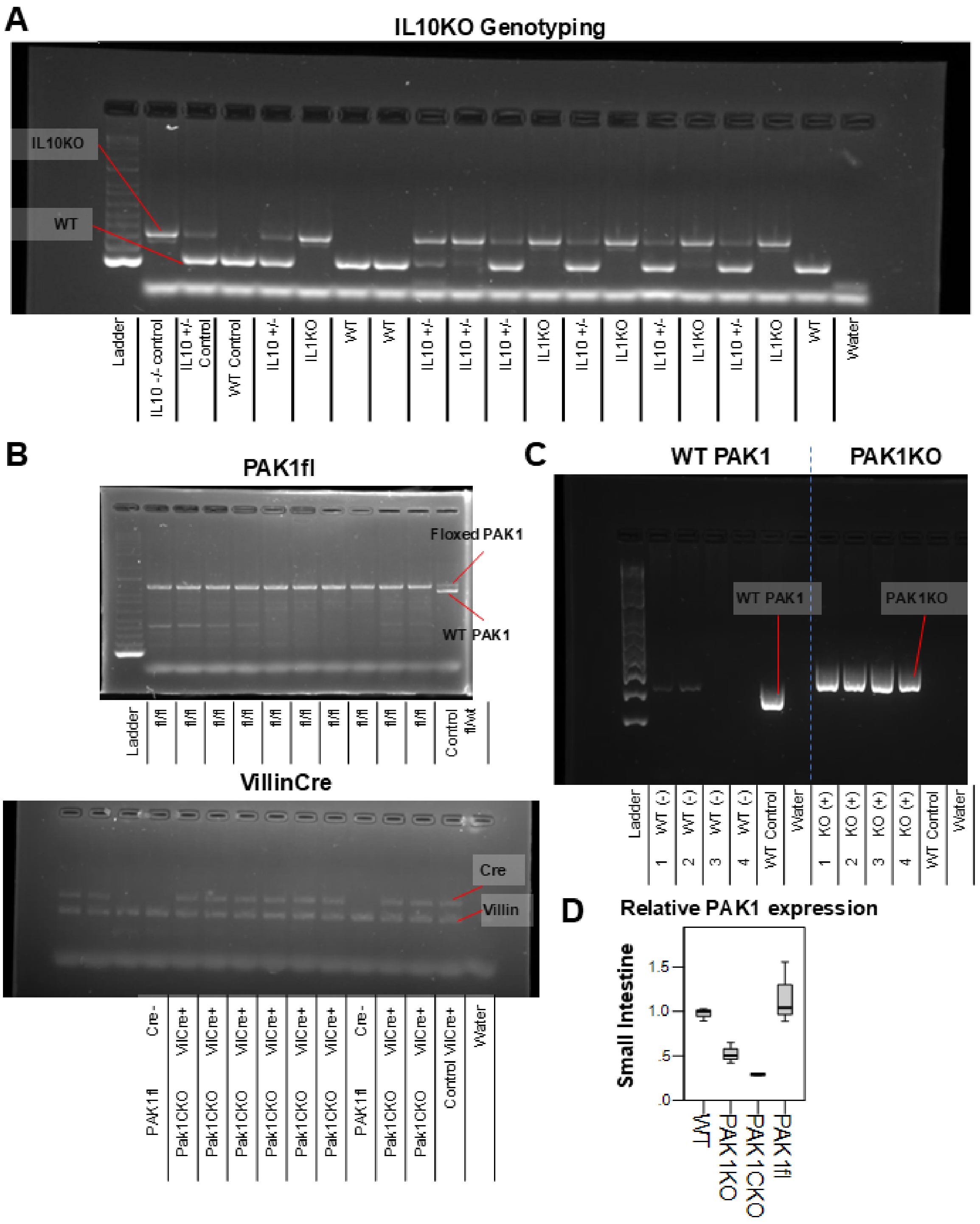

Supplementary Figure 3. Genotyping for PAK1KO and IL10KO. A. Representative PCR blots from tail genotyping for IL10KO and WT in mice from the IL10 x PAK1CKO crossbreeding. B. Representative PCR blots from tail genotyping for floxed PAK1 and VillinCre and C. PAK1KO. PAK1KO in separate blots as protocol involves separate PCR runs for wild-type PAK1 and PAK1KO. D. Relative expression of PAK1 in whole small intestinal lysates from untreated animals WT (n=3), PAK1CKO (n=2), PAK1fl (n=3). Normalized to Actin.

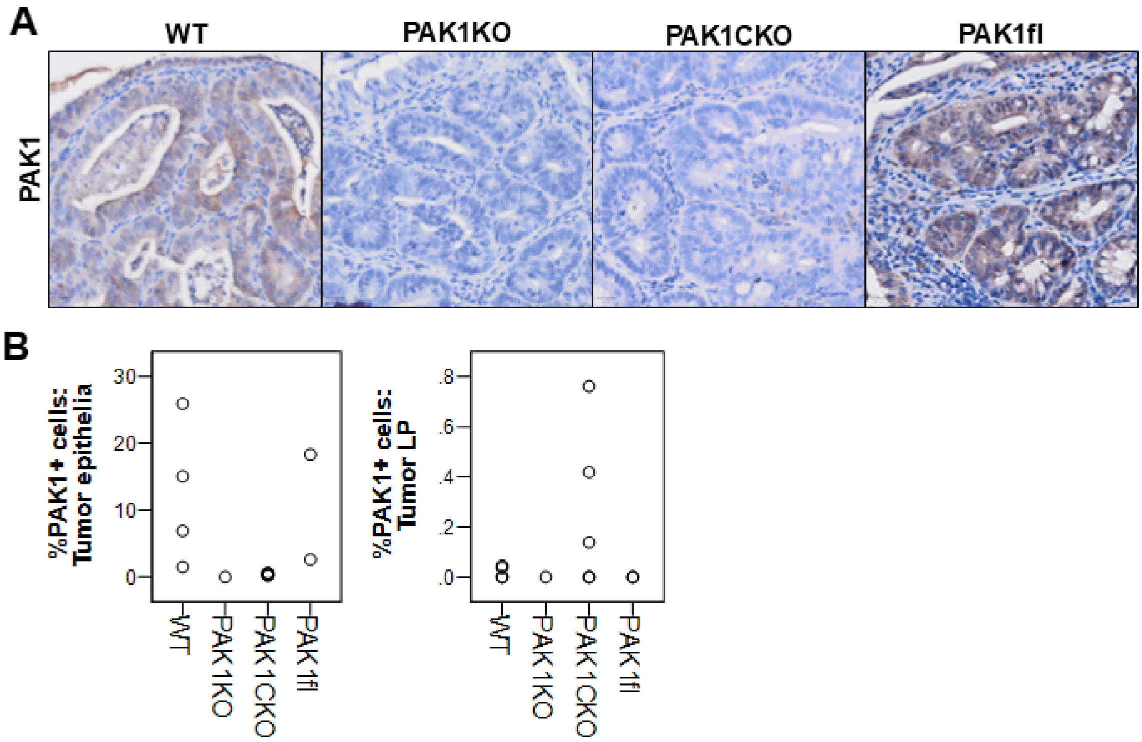

Supplementary Figure 4. PAK1 expression in intestinal tumors. A. Representative images of PAK1 staining in colonic tumors. B. Mean percentage of PAK1 positive cells in epithelia and lamina propria (LP)

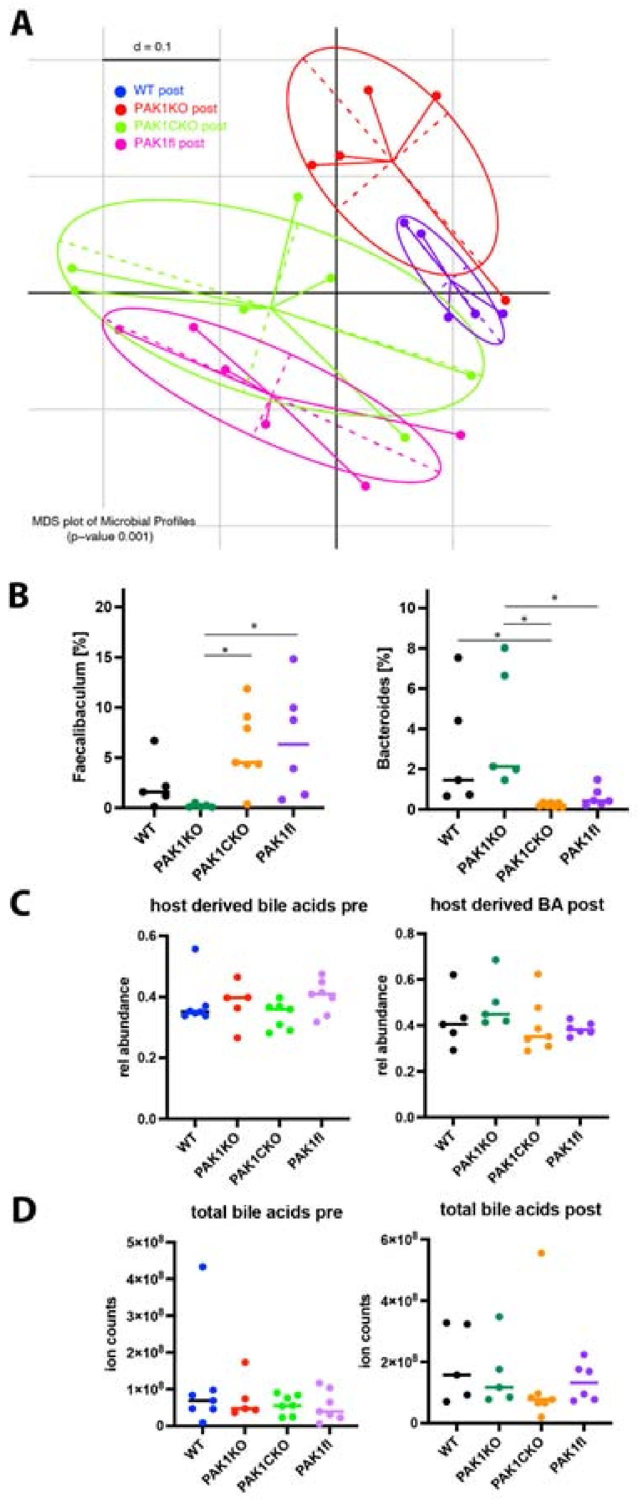

Supplementary Figure 5. Microbial profiles post-AOM/DSS treatment and bile acid analysis. A. NMDS plot of fecal microbial profiles after AOM/DSS treatment (post-) B. Differentially expressed genera in post- samples of floxed PAK1 versus WT or PAK1KO mice. C. Bile acid composition in fecal samples collected pre- and post-AOM/DSS. WT (n=5), PAK1KO (n=5), PAK1CKO (n=7) and PAK1fl (n=6) *≤ 0.05
